# Medical Items and News of Interest to the Physician

**Published:** 1874-10

**Authors:** 


### Medical Items and News,

### For the Use of Physicians.

CULLED FROM THE STANDARD MEDICAL JOURNALS OF THE WORLD.

NOTE.—These formulæ are intended only for the regular practitioner of medicine, and should be employed only by him, or under his immediate supervision.

**Antidote for Phenic Acid.**

Lime sucrate is the antidote recommended.

**Gelseminum in Rheumatism.**

Gelseminum, in doses of from five to thirty drops, repeated at intervals, until the pain and other inflammatory symptoms disappear, is most serviceable in inflammatory rheumatism.

**Incontinence of Urine of Old People.**

A lady of eighty years of age was entirely relieved by the continued use of tincture of iodine, (one to six drops a day). This treatment is not new.—*Allg. Med. Zeitschr.*

**Diabetes.**

Dr. O. Shultzer claims great success in the treatment of diabetes under the free use of glycerine, internally, with citric acid and abstinence from starchy food.—*New Remedies.*

**Headache after Drunkenness.**

Byron recommends "sermons and soda water." The *Revue de Therapeutique* says: Take of solution of acetate of ammonia, tincture of bitter orange peel, syrup of bitter orange peel, each 20 parts; water 500 parts. To be given in repeated tablespoonful doses.

**Iodine Caustic.**

Is prepared by dissolving four grammes of iodine in eight grammes of glycerine. It is used in lupus by applying it once every other day, and covering the parts with gutta-percha. This treatment is continued for several weeks.

**Pneumonia.**

A writer in the *Lancet* recommends strongly the use of arnica in pneumonia, in doses of ten minims of the strong tincture every two hours. The pulse should be reduced by it to 60 or 70, and it descends at times as low as 40 per minute. The relief is immediate and marked.

**Freckles.**

It is said that powdered nitre moistened with water, applied to the face night and morning, will soon remove freckles.—*Practitioner and Druggist.*

**Agreeable Purgative.**

℞.

Magnesiæ calcinat......... ʒ jss.

Aquæ puræ................ ℥ ij.

Syrup orgeat (or curacoa).. ℥ ss. M.

—*Boston Med. & Surg. Reporter.*

**Diphtheria.**

In diphtheria, M. Bucquoy employs locally lemon juice. He saturates cotton wool with the juice, and presses it against the affected surface four or five times a day.—*Jour. Applied Chemistry.*

**Freckles.**

Sulpho-carbolate of lime...... ʒ ij.

Glycerine.................. ʒ xxv.

Rosewater.................. ʒ xxv.

Alcohol.................... ʒ v.

Mix. Anoint the skin twice daily, letting it remain damp with the lotion half an hour.

**Compound Syrup of Garlic.**

℞.

Fluid ext. allii............. ʒ ij.

Sprupi scillæ............... ʒ iv.

Tinct. opii comp .......... ʒ ij.

Syrupi simplicis........... ℥ ij.

M. A teaspoonful is a dose for a child three or four years old, for catarrh.

**Toothache Drops.**

℞

Chloral.................. ʒ i.

Camphor.................. ʒ i.

Morphia.................. grs. ij.

Oil of pepermint........... ʒ ij.

Mix.

**For Caseous Pneumonia.**

℞.

Acidi citrici ............ ℈ i.
Potassii cyanidi.......... grs. v.
Morphiæ sulph............ grs. ij.
Tinct. hyosciami ......... ʒ i.
Syrupi tolutani........... ℥ iv.
Syrupi picis comp......... ℥ ij.

Sig. Take a teaspoonful every third hour. From Dr. J. W. Robbins, of Philadelphia.

**Hæmorrhoids.**

Dr. Wm. Colles, Dublin, lately injected twenty minims of tincture perchloride of iron into each internal hæmorrhoidal tumor. No traces could be found afterward, by speculum, except nodules, of the size of shrivelled currant. The case had resisted Dr. Huston's application of fuming nitric acid.—*British Med. Journal.*

**The Sovereign Balm for Syphilis.**

℞.

| | |
|---|---|
| Hydrarg, iodidi vir ........ | grs. xij. |
| Ext. cannabis ind......... | grs. vi. |
| Ext. conii..... ......... | grs. xij. |
| Lupulinæ ................. | grs. xij. |

M. Div. in pil. No. xij. Sig. One pill thrice a day.

**Valuable Formula in Cerebro-Spinal Meningitis.**

℞.

| | |
|---|---|
| Potassii bromidi ........... | ʒ ij. |
| Fl. ext. ergot.............. | ʒ iij. |
| Tinct. belladonnæ ......... | ʒ i. |
| Tinct. aconiti rad.......... | gtt xij. |
| Curacao cordial, q. s. ad ... | ℥ iij. |

M. Sig. Dessertspoonful every three hours.

**Puerperal Convulsions.**

℞.

| | |
|---|---|
| Potassii bromidi........... | ʒ i. |
| Chloral hydrat ............ | ʒ i. |
| Camphoræ ................. | grs. vi. |
| Tinct, card. comp ...... | ʒ vi. |

M. Sig. Take a dessertspoonful every half hour until relieved.

**Jalap Biscuits.**

M. Tambureau, a pharmacien residing at Guelma, Algeria, publishes the following directions for the preparation of biscuits containing the resin of jalap, which he says are of good appearance, and have an agreeable taste:—

| | | |
|---|---|---|
| Pure jalap resin........ | 56 | grammes. |
| Powdered sugar and flour | 1000 | " |
| Tincture of vanilla...... | 10 | " |
| White of egg...........No. 20 | | |
| Yolk of egg............No. 40 | | |

Emulsify the resin with the egg yolks, add successively the sugar, tincture and flour, and make a homogeneous paste, into which thoroughly incorporate the egg-whites previously beaten up. Divide the mass into 144 biscuits.

**Bichloride of Methylene.**

Dr. B. F. Dawson, of New York, claims for this anæsthetic, in comparison with ether and chloroform, that it is pleasanter in odor, more rapid in effects, induces no muscular excitement, recovery is more rapid and complete, nausea and vomiting are rarely induced by it, it is not so depressing and respiration is more regular and free; requires but a small amount. —*Medical Record.*

**Menorrhagia.**

Dr. J. K. Black, Newark, O., in the Cincinnati *Lancet*, suggests:

℞.

| | |
|---|---|
| Ammonii bromidi......... | ℥ j. |
| Syrup aurant. cort. | |
| Aquæ..................... | āā. f. ℥ iij. |

M. Sig. A teaspoonful before tea and bedtime, commencing ten days before the expected period.

**Pain in Bowels.**

For the intense pain of cholera morbus, etc., liable to prevail at this season, the following is useful:

℞.

| | |
|---|---|
| Chloroformi .............. | f. ʒ jss. |
| Morph. sulph ............ | gr. j. |
| Tr. opii. comp. | |
| Tr. cardam. comp.......... | āā f. ℥ ss. |
| Syr. zingib. .............. | f. ℥ j. |

M. Sig. Teaspoonful every 15 minutes till relieved.—*Med. & Surg. Reporter.*

**Chloral as an Anæsthetic during Labor.**

Dr. W. Playfair (*The Lancet*, Feb. 21, 1874) has found that chloral has the immense advantage over chlorform, when administered during labor, of not lessening the strength or intensity of the pains, while at the same time markedly diminishing the suffering resulting from them. It is chiefly applicable at a period when we would not think of administering chloroform—towards the termination of the first stage of labor, before the complete dilatation of the os, and when the sharp, grinding pains perhaps produce more suffering and are less easily borne than the more forcing pains of a later stage.

He gives the drug at first in fifteen grain doses, and then in smaller quantity; increasing the intervals between its administration, and thus usually keeps up a full and sufficient effect for hours. It need not at all interfere with the exhibition of chloroform.

**Lotion for Fetid Feet.**

*L'Union Medicale* gives this recipe :—

Permanganate of potash fifteen parts, distilled water 1000 parts. The feet to be washed twice a day with the lotion. They are then to be carefully dried and powdered, either with potato-starch or lycopodium.

---

**Cholera Morbus.**

℞

| | |
|---|---|
| Magnesiæ ust .............. | ʒ i. |
| Morphiæ .................... | gr. i. |
| Pil. hydrarg................. | grs. viij. |

Divide into four powders and give one every half-hour until relieved.

---

**Diphtheria.**

M. Revillout, in the *Gazette des Hopitaux,* recommends in the strongest terms the employment of large quantities of pure lemon-juice as a gargle. He says that he and his father have used it during eighteen years, and always with success, it being the most certain application yet known.

---

**Uterine Neuralgia.**

℞.

| | |
|---|---|
| Tinct. aconiti rad. (Fleming) | ʒ iss. |
| Ammonii chloridi.......... | ʒ ij. |
| Ammonii iodidi ........... | ʒ i. |
| Tinct. card. comp.......... | ℥ i. |
| Syrupi aurant............. | ℥ iv. |
| Aquæ anisi q. s. ad ........ | ℥ viij. |

M. Sig. Teaspoonful every four hours. Also give half an hour before each meal, a teaspoonful of the syrup of the phosphates of iron, ammonia, quinia and strychnia. This treatment seldom fails to give relief after all other means have failed.

---

**Neuralgia of the Hip and Thigh (Sciatica).**

℞

| | |
|---|---|
| Chloral hydrat.............. | ʒ iij. |
| Sodii bromidi............... | ʒ iij. |
| Morphiæ sulph............. | grs. vi. |
| Quiniæ sulph............... | grs. liv. |
| Liq. arsenici chloridi........ | ʒ ij. |
| Pulv. camphor.............. | ʒ ss. |
| Elix. taraxac. comp.......... | ℥ vi. |
| Tinct. aconiti............... | gtt. xxiv. |

Mix. Take one dessert-spoonful every three hours until relieved. This compound has given entire relief in ten cases and failed in three.

**Rheumatic Liniment.**

℞

| | |
|---|---|
| Chloral ..................... | ʒ iv. |
| Camphor.................... | ʒ vi. |
| Tinct. aconite root........... | ℥ i. |
| Oil cajeputi ................. | ʒ iij. |
| Alcohol, sufficient to make.... | ℥ iv. |

Apply with camel's-hair pencil over the painful part.

---

**Acetate of Lead Lotion.**

M. Bouchut (*Gazette des Hopitaux*) mentions a case where a lady had used a lotion called "Eau de Mad. Delacour," a favorite quack remedy in Paris for sore nipple. She had omitted to wash the lotion off before putting the child to the breast. It was seized with violent colic, and died in a few days with all the usual symptoms of lead-poisoning.

---

**Balsamic Cigarettes for Asthma, etc.**

The London *Chemists' and Druggists' Compendium* gives the following recipe :—

Soak strong unsized paper in a solution of saltpetre ; this dry, and treat first with tincture of cascarilla, and afterwards, when nearly dry, with compound tincture of benzoin ; cut into squres of a suitable size, and roll into the form of cigarettes.

---

**Hair Tonic.**

℞

| | |
|---|---|
| Adipis...................... | ℥ ij. |
| Ceræ albæ.................. | ℥ i. |
| Bal. Peruvian ............... | ʒ ij. |
| Tinct. canth................. | ʒ ss. |
| Olei lavendulæ.............. | gtt. xvi. |

Melt the lard and wax together by gentle heat ; add the other ingredients and stir until cold. This preparation has no superior in restoring the growth of the hair.

---

**To Administer Creosote.**

As creosote is now frequently employed in the treatment of typhoid fever, and is exceedingly distasteful to some patients, the *Canada Medical Journal* gives the following formula which in great measure covers its flavor, and is easily prepared:—

| | |
|---|---|
| Creosote................... | 3 drops. |
| Essence of lemon......... | 2 drops. |
| Orange-flower water...... | 1 oz. |
| Spring water.............. | 3 oz. |

A spoonful to be taken at frequent intervals throughout the day.

**Intermittent Fever.**

℞.
Quiniæ .................. grs. xvi.
Hydrarg. sub. chloridi..... grs. viij.
Podophylli resin........... gr. ss.
Gelsemini (Keith's)........ gr. iss.

M. Div. in pil. No. viii. Sig. One pill every hour until all are taken.

**Liniment.**

℞.

Tinct. aconiti rad. sati...... ℥ i.

Chloroformi.............. ℥ i.

Camphoræ............... ℥ i.

Rhigolene................ ℥ ss.

Liq. saponis ad............ ℥ iv.

M. Sig. Apply as required with a camel's hair pencil.

**A Diaphoretic.**

℞.
Liq. ammoniæ acet ........ ℥ vi.
Spirits ether nitrosi........ ℥ ss.
Ant. et potassæ tart........ gr. i.
Morphia .................. gr. i.
Syr. acidi citrici ........... ℥ ss.

M. Tablespoonful every three hours.

**Elixir Chloroforn---Useful in Colic.**

℞
Chloroform.
Tinct. Opii.
Tinct. Camphoris.
Spir. Ammon. Aro......āā. ℥ iss.
Oil Cinnamon.......... gtt. xx.
Brandy................ ℥ ij.—M.

Sig.—Half a fluid drachm, more or less.—*American Eclectic Medical Journal.*

**Styrax in Itch.**

In the Catharine Hospital, in Stuttgard, they treat scabies with the following ointment :

℞.
Styracis.................. ℥ j.
Olei olivæ.
Spir- vin. com............. āā ʒ j. M.

The patient, if an old case, is first washed thoroughly with soft-soap, having it well rubbed in, nine to twelve times in three days, and then anointed with the above one to three times in one day. In recent cases the soft-soap treatment is not required. In 1659 cases thus treated in three years, every one was cured, and although no precautions were taken to destroy the insects on their clothing, *not one relapse occurred.*

**Chronic Bronchitis.**

℞.

Copaibæ................. ʒ iv.

Liq. Potassii............. ʒ i.

Pulv. ext. glycyrrhizæ..... ʒ ij.

Syrup. scilæ.............. ℥ i.

Ol. amygdalæ amar ....... ♏ x.

Paregoric ................ ℥ i.

Syrup of acaciæ q. s....... ℥ iv.

M: Teaspoonful every three or four hours.

**Almost a Specific in Dysentery.**

℞.

Pil. hydrarg............... grs. xij.

Pulv. ipecac............... grs. xij.

Gelsemini................. grs. ij.

M. Divide in 12 pills. Give one every three hours until the effect of the gelseminum is manifest in debility of the eyelids, and then discontinue. This treatment seldom fails to arrest at once the violence of the disease.

**Liquor Sedativus.**

This is a combination often prescribed by English physicians in diseases complicated with febrile symptoms:—

Tinct. Opii Camphor.
Spts. Æth. Nit. dulc.
" Mindereri.
Syr. Simpl.
Aq. Camphoræ, āā part. æq.

M. et ft. solutio. Dose: a teaspoonful.

To increase the therapeutic effect of this mixture, 2 fl. ʒ of Tinct. Gelsemini or 1 f. ℥ of Tinct. Verat. vir. are often added to four ounces, to meet particular indications.

**Acute and Subacute Rheumatism.**

The leading feature in the treatment of these cases in the Roosevelt Hospital, consists in the administration of iodide of potassium in doses varying from 8 to 15 grains, combined with 15 drops of the wine of colchicum-seeds, three times a day. To relieve the local pain about the joints, the application of ice in ice-bags has proven most satisfactory. When the acute symptoms have been subdued, or have subsided, the application of kerosene oil to the stiffened joints, accompanied with free rubbing of the parts, has afforded more relief than other applications. Whether the virtue is in the oil, or in the rubbing, or in both, can hardly be stated, but the beneficial results which have been derived from its use warrant its adoption.

**Prurigo and Pruritus.**

Dr. Rothmund (*Ærztl. Intelligenzblatt*, 39, 1872) states that the internal administration of carbolic acid in pruritus excels every other method. He has tried also the hypodermic injection of it with marked success, there being no local irritation produced, as one would expect beforehand. Solutions of pure carbolic acid seem to be more efficacious than those of carbolate of soda.—*The* (*London*) *Medical Record,* Jan. 22, 1873.

---

**To Deprive Iodine of Stain.**

Add a few drops of phenic acid to the tincture, and it will not stain ; moreover, the tincture is more efficacious, and its action more certain. M. Bogs recommends the following formula for use in injections :

Alcoholic tincture iodine... 3 grm.
Phenic acid ............... 6 drops.
Glycerine.................. 30 grm.
Distilled water.............150 grm.

—*Med. and Surg. Reporter.*

---

**To Check Vomiting from Cough.**

Consumptives and others suffering from paroxysms of cough frequently vomit their food in such paroxysms. Dr. Woilliez recommends swabbing the larynx before eating with a concentrated solution of bromide of potash :—

℞

Pot. brom.................. ʒ ij.

Aquæ destil................ ʒ iv.

Warning the patient not to cough for a few minutes after the application. The same treatment is recommended for the vomiting of pregnancy. It is said to be very successful.

---

**Irritable Heart.**

Dr. Da Costa, in speaking of the treatment of irritable heart occurring in soldiers, with hypertrophy, states that no medicine can be compared to aconite. In moderate doses, used for months, a decrease of the enlarged organ happened. In pure irritable heart he recommends digitalis, digitaline, and veratrum viride—the latter stands between aconite and digitalis. Ten drops of the tincture of digitalis were given three times daily, and from 1-60th to 1-30th of a grain of digitaline, to some of his soldier-patients. He is disappointed in gelseminum as a cardiac sedative. In instances of irregular action, belladonna and atropia proved very efficient agents.—*American Journal of the Medical Sciences.*

**Facial Neuralgia.**

In *The British Medical Journal*, Dr. F. B. Lee reports the case of a lady, æt. 32, who had suffered for years from attacks of facial neuralgia, had had several teeth extracted, had been blistered behind the ears, and had tried numerous other means without avail. He prescribed croton-chloral in three-grain doses every four hours. After the third dose perfect ease was experienced, and, although three months had elapsed, there had been no return of the disease.

---

**Tamarind Cough Elixir.**

The London *Chemist and Druggist* gives this formula in reply to the question of a correspondent :—

Boil gently half a pound of West Indian Tamarinds in a pint of distilled water for half an hour. Cool and strain, make up with more distilled water to the bulk of a pint, and add :—

Acet. scillæ................ ʒ x.

Acid. acet. dil.............. ʒ iv.

Tinct. Camph. co..........

Æther. chlor...............āā. ℥ ijss. M.

A teaspoonful for a dose.

---

**Seborrhea.**

Mr. Canty, of Liverpool, in his late work on "Cutaneous Medicine," recommends as the best solvent for sebum, benzole, used either pure or as a lotion. "If," he says, "there are collections in the convolutions of the ear, where instruments can only be inconveniently used, pure benzine, applied with a brush, will readily dissolve the sebum, leaving the central black spot quite intact, as a pillar of dirt and hairs in a cavity. If used on the face, back, or shoulders, some camphorated oil being added to ten times the quantity of almond oil. The mixture should be rubbed into the skin at night. In the morning the benzole lotion should be applid with a sponge or flannel, and afterward the parts well washed with warm water and soap." He gives the following formula :—

℞
Benzole...................... ℥ ss.
Gum tragacanth............ ℥ ss.
Aquæ........................ ℥ viij.

We need not add that comedones or pimples on the face, so annoying to many young persons, are forms of seborrhea.—*Med. and Surg. Rep.*

**Syrup of Borax.**

The following is commended by the *Cincinnati Lancet* for laryngeal catarrh :—

| | |
|---|---|
| Borax | 4 dr. |
| Syrup simp | 10 oz. |

Dissolve by the aid of heat. A teaspoonful to be taken from 7 to 10 times a day, and swallowed without dilution. Avoid drink immediately after, so as to prolong the contact of the remedy.

---

**Tape-Worms.**

When the anæsthetic power of ether was first discovered, it was only proposed to use it on human beings to render surgical operations painless. Von Heydon, the merciful man who would not inflict pain on any living creature, employed it as long ago as 1830 for killing insects for his collection. Even worms were rendered dormant and helpless by its use. Prof. August Vegel now announces a new application of this anæsthesia for worms —its application to tape-worms. The ether is inclosed in a galatin capsule and swallowed. The ether is vaporized in the stomach and the worm stupefied, it being then easily removed by any of the usual remedies, against which, when awake, the worm offers a strong resistance.

---

**Emulsion of Raw Meat.**

We quote from the *Repertoire de Pharmacie* a formula for the above, which was given by its inventor (M. Yvon) at a meeting of the *Societe d'Emulation pour la Science Pharmaceutique*. The object was to provide an agreeable means of administering raw meat, a remedy much in fashion with some of the Continental physicians. M. Yvon takes

| | | |
|---|---|---|
| Raw meat | 250 | grammes. |
| Sweet almonds | 75 | " |
| Bitter " | 5 | " |
| White sugar | 80 | " |

The almonds are blanched and the whole beaten up in a marble mortar until a rose-colored homogeneous paste is obtained. This is said to be a very pleasant flavor and readily taken by sick persons. It may easily be made into an emulsion with water, which will not unmix for twenty-four hours; the emulsion can be made still more nourishing by the addition of the yolks of two eggs and by being made up with milk instead of with water.—*Chemist and Druggist.*

**Guarana in Chronic Rheumatism.**

Dr. Edward A. Rawson, Assistant Surgeon to the Carlow Infirmary, writes to the *Irish Hospital Gazette*, April 15th:—

Suffering severely from lumbago, and finding all vaunted remedies fail, I tried guarana as an experiment. I took fifteen grains blended with hot water, and added cream and sugar. For twenty-four hours afterwards I had a delightful relief from pain. I thought it must be a coincidence; but, on a return of the lumbago, took another dose in the same manner and with a similar result. I gradually increased the dose to forty grains, and took it regularly, once a day, for about a week. The lumbago disappeared. I gave up the guarana, and in a few days the pain in the back returned. A forty-grain dose removed it, and it did not return for several days afterwards. Now, whenever it does, I have my remedy at hand. During the last month I have experimented largely with guarana on a variety of patients, rich and poor. The results vary. When the pain is acute, coming on with sharp stings, guarana acts like magic; when it is of a dull, aching character, the drug is slower in its action, and several doses must be taken before any decided benefit can be perceived.

I have come to the following conclusion, viz: that whenever the fibrous envelopes of nerves, the aponeurotic sheath of muscles, the fasciæ or tendons are the parts affected, guarana gives, if not instantaneous, at least very immediate relief, which will last from twelve to twenty-four hours; and I confidently expect that perseverence in the use of the drug, gradually increasing the dose up to forty grains, will entirely remove any of the above mentioned kinds of rheumatism.

Of the good effects of guarana on nervous hemicrania there is no doubt; and I trust it will prove, in other hands, as valuable against rheumatism as it has in mine.

I find guarana was examined by Martius, in 1829, and by Gravelle in 1840. According to them "it stimulates, and at the same time soothes, the gastric system of nerves, and reduces the excited sensibility of the cœliac plexus, thereby diminishing febrile action, and strengthening the stomach and intestines, particularly restraining any excessive mucous discharges; at the same time increasing the action of the heart and arteries, and promoting diaphresis."—*Med. and Surg. Reporter.*

**Carbuncle Treated by Carbolic Acid.**

Dr. Peter Eade writes, in the London *Lancet*, that he has found the action of carbolic acid in carbuncle constantly beneficial. He uses one part of acid to four or five of oil or glycerine. He thinks the efficacy of the solution is limited almost absolutely to those parts with which it could be brought into actual contact; and, although it appears occasionally to have produced injurious effects when used in large quantity, he has kept a large sloughing and granulating surface, for days together, constantly covered by the carbolized oil without any harm arising, although the urine soon presented the peculiar blackish color which has been several times observed during its employment.

---

**Cod-Liver Oil.**

Will you allow me to add, in the *Reporter*, one more to the many formulæ for the administration of that excellent remedy, cod-liver oil. Why have so many physicians become sceptical about the virtue of cod oil? Why so many say they place no confidence in it? Because they do not properly prescribe it. It must be emulsified before administration, to produce its legitimate effect. Indeed, I believe if it is not emulsified before it is taken into the stomach, that in very many instances it is not at all.

Is it not a noticeable fact, that phthisical patients, aye, even individuals with a hereditary, or latent tendency towards tubercular disease, almost entirely eschew fats, discard them from their diet table? Are they not intensely averse to castor-oil as a cathartic? Do we not see people dying of consumption, and ask, "Have you thoroughly tried the oil?" and they make doleful answer, "Yes," so many bottles. Perhaps a dozen, without apparent benefit. Clear, unemulsified, unassimilated oil. Almost as well to have poured it in their boot-leg for digestion and nutrition. And is there cause for this? We probably all accept as true the assertion of physiologists, that one of the chief functions of the pancreatic juice is to emulsify fats. Can the pancreas be at fault? If it is, aid it.

I think it is Headland, in his essay on the action of medicines, than which there is no better, who claims ether to be a pancreatic stimulent. We might say, with delightful Capt. Cuttle, when read make a note of, for we believe Headland to tell very few idle tales:—

℞

| | |
|---|---|
| Cod-liver oil | fl. ℥ ix. |
| Saccharated pancreatin | gr. ccixx. |
| Acid phos. dil. | fl. ℥ iv. |
| Fl. ext. sweet orange peel | fl. ℥ ss. |

Or

℞

| | |
|---|---|
| Cod-liver oil | fl. ℥ vi. |
| Saccharated pancreatin | gr. clxxx. |
| Muriatic acid | fl. ℨ ss. |
| Fl. ext. sweet orange peel | fl. ℥ ss. |
| Aquæ | fl. ℥ xi. |

Put the pancreatin and acid (and water) into a pint bottle and shake thoroughly, then add the oil and agitate, lastly, add the fluid extract. Very thorough agitation, both before and after the addition of the oil, is quite essential.

The latter formula is that of Mr. J. S. Plumb, a pharmacist, of Syracuse, New York, who asseverates that it makes a pretty agreeable mixture. I prefer the former, because of the increase in amount of oil, the substitution of phosphoric for muriatic acid, and the expulsion of a considerable amount of inert water. The emulsions are equally good, and one dessertspoonful of either is worth a tablespoonful of clear oil. The pancreatin recommended is that of Dr. Wm. Manlius Smith, of Syracuse, an unquestionably reliable article, of which thirty grains, well diffused in an ounce of water, will emulsify one ounce of cod oil.—F. C. Clarke, M. D., in the *Med. and Surg. Reporter*.

---

**Nicotine an Antidote to Strychnia.**

A case of poisoning by strychnia which was successfully treated with nicotine, has been published in the *British Medical Journal*, by the Rev. Dr. Houghton, F. R. S., of Trinity College, Dublin. When the treatment commenced, the patient, a lad nineteen years of age, was violently convulsed, his pupils were dilated, and his arms and legs were rigid. The nicotine was administered in drop doses in whisky-punch every half-hour. After the second dose the paroxysms were less violent; and when he had taken four doses he was much better, and eventually he recovered. The poisoning was caused by the lad picking up and eating an egg which had had strychnia introduced into it, and been placed in a garden for the purpose of poisoning magpies.—*Pharmaceutical Journal.*

